# The role of ratio markers based on prealbumin in the diagnosis of periprosthetic joint infection

**DOI:** 10.3389/fcimb.2025.1597401

**Published:** 2025-07-08

**Authors:** Qianqian Cao, Xinchuang Ning, Panlong Fan, Tianmiao Cheng, Yuzhe Zhang, Cheng Cheng, Zhipeng Dai

**Affiliations:** ^1^ Department of Orthopaedics, Zhengzhou University People’s Hospital, Henan Provincial People’s Hospital, Zhengzhou, Henan, China; ^2^ Department of Orthopaedics, Xinxiang Medical University, Xinxiang, Henan, China; ^3^ Center of Clinical Laboratory, The First Affiliated Hospital of Soochow University, Suzhou, Jiangsu, China; ^4^ The First Laboratory of Cancer Institute, The First Hospital of China Medical University, Shenyang, Liaoning, China

**Keywords:** periprosthetic joint infection, prealbumin, aseptic loosening, marker, diabetes

## Abstract

**Background:**

Periprosthetic joint infection (PJI) is a severe complication following total joint arthroplasty, and the timeliness of its diagnosis and treatment is crucial for patient recovery. Although various biomarkers have been extensively evaluated and applied in clinical practice, the diagnosis of PJI remains challenging. Therefore, it is necessary to identify more precise biomarkers for the diagnosis of PJI. This study aims to investigate the value of ratio-based biomarkers using prealbumin (PA) for the diagnosis of PJI.

**Methods:**

This study compared the levels of C-reactive protein (CRP), erythrocyte sedimentation rate (ESR), fibrinogen (FIB), PA, CRP/PA (CPR), ESR/PA (EPR), FIB/PA (FPR), and the combined ratio of CPR+EPR+FPR(CEF) in 180 patients with PJI and 105 patients with aseptic loosening (AL) who presented at our department from January 2019 to December 2024. The diagnostic efficacy of these markers in PJI diagnosis was assessed using the area under the curve (AUC) of the receiver operating characteristic (ROC) curve.

**Results:**

Among these biomarkers, CPR has the highest AUC of 0.921 (95%CI 0.890-0.952), and it can distinguish PJI with a cut-off value of 0.0366, a sensitivity of 76.1%, and a specificity of 95.2%. In the diabetic subgroup, the combined biomarker CEF has an AUC of 0.951 for diagnosing PJI, with a sensitivity of 88.9% and a specificity of 94.4%.

**Conclusion:**

Ratio-based markers based on PA show promise as valuable new adjunctive diagnostic markers for PJI.

## 1 Introduction

Periprosthetic Joint Infection (PJI) is one of the most destructive complications following hip and knee arthroplasty and is also a major cause for patients to undergo revision surgery (Sarokhan et al., 1983; Koh et al., 2017; Schwartz et al., 2020). Related studies have reported that the incidence of PJI after total joint arthroplasty is 2%-2.4% in the United States and 0.33%-1.14% in China (Kurtz et al., 2012; Peng et al., 2019). PJI not only significantly prolongs patients’ hospital stays and increases medical costs, but also significantly raises the risk of long-term disability (Xu et al., 2021). Therefore, early, rapid, and accurate diagnosis of PJI is crucial to mitigate these negative impacts. Currently, the results of pathogen culture are still considered the “gold standard” for diagnosing PJI (Gazendam et al., 2022). However, the presence of bacterial biofilms greatly reduces the detection rate of pathogens, which increases the difficulty of diagnosing and treating PJI (Yoon et al., 2017; Koutserimpas et al., 2022). In recent years, researchers have advocated the use of polymerase chain reaction, mass spectrometry, and next-generation sequencing as new methods for diagnosing PJI (Esteban and Gómez-Barrena, 2021; Tan et al., 2022). These emerging technologies can significantly improve the diagnostic accuracy of PJI, but they are difficult for most medical institutions to perform independently and are associated with higher detection costs (Torchia et al., 2019).

Peripheral blood testing is a routine examination for inpatients due to its simplicity, cost-effectiveness, and efficiency, and it is widely used for the diagnosis of various diseases (Deng et al., 2024). In clinical practice, C-reactive protein (CRP) and erythrocyte sedimentation rate (ESR) have been extensively used for the diagnosis of PJI. However, due to their relatively low sensitivity, these two biomarkers are not ideal for ruling out PJI. Therefore, there is still a need to further explore new biomarkers for diagnosing PJI, whether they are single biomarkers or combinations of biomarkers, in order to identify more precise indicators and thereby significantly improve the accuracy of PJI diagnosis.

Prealbumin (PA) is not only a nutritional biomarker commonly used to assess the nutritional status of patients, but also a negative acute-phase protein during infection (Sneh et al., 2020). As a positive acute-phase protein, fibrinogen (FIB) is closely related to the state of infection (Chandy et al., 2017). Previous studies have confirmed that FIB has good diagnostic efficacy for PJI (Song et al., 2023). In recent years, studies have reported that the ratio of C-reactive protein to prealbumin (CPR) and the ratio of fibrinogen to prealbumin (FPR) have significant clinical value in the diagnosis and prognosis assessment of inflammatory and neoplastic diseases (Wang et al., 2020; Ying et al., 2021; Maruyama et al., 2022; Guo et al., 2025). Yu et al. found that CPR outperforms traditional inflammatory markers such as ESR in diagnosing active pulmonary tuberculosis (Yu et al., 2023). Meanwhile, Ying et al. found that FPR can effectively distinguish early colorectal cancer from colorectal polyp subgroups, identify high-risk stage II colorectal cancer patients, and provide a basis for selecting appropriate treatment plans (Ying et al., 2022). However, no studies have yet explored the application value of CPR and FPR in the diagnosis of PJI. In addition, the potential value of the ratio of ESR to PA (EPR) in diseases has not been explored. Previous studies have reported that the incidence of PJI after primary joint replacement in diabetic patients is significantly increased (Jämsen et al., 2012; Wier et al., 2024). We speculate that diabetes may have an impact on the diagnostic capacity of markers for PJI. Therefore, this study retrospectively analyzed the serological data of patients in our hospital for the first time to explore the diagnostic efficacy of PA, CPR, FPR, EPR, and the combined use of CPR+EPR+FPR (CEF) for PJI and further investigated the diagnostic value of these new biomarkers for PJI in the diabetes mellitus subgroup. Our hypotheses are as follows: (i) Compared with AL patients, the levels of CPR, FPR, EPR, and CEF will exhibit elevation in PJI patients; (ii) In the detection of PJI, CPR, FPR, EPR, and CEF will show comparable diagnostic efficacy to ESR, CRP, and FIB.

## 2 Patients and methods

### 2.1 Study design

This study is a single-center retrospective investigation, which included the medical records of patients who underwent revision arthroplasty at our hospital from January 2019 to December 2024. Cases diagnosed with PJI and Aseptic Loosening (AL) were selected. This study included patients of all ages who met the criteria. Detailed records were kept of the patients’ age, gender, surgical site, comorbidities, as well as the levels of CRP, ESR, FIB, and PA in the early morning of the first day after admission. By collecting relevant patient data, this study aims to systematically evaluate the efficacy of CRP, ESR, FIB, PA, CPR, FPR, EPR, and CEF in PJI. On this basis, the study further focuses on the patient subgroup with diabetes mellitus, delving into the diagnostic value of the aforementioned indicators for PJI in this population. It is hoped that this will provide more targeted references for clinical precision diagnosis. This study strictly adheres to the ethical principles of the Helsinki Declaration regarding human medical research and has been approved by the Ethics Committee of the People’s Hospital of Henan Province.

### 2.2 Definitions of PJI and AL

As shown in **Table 1**, the diagnostic criteria for PJI are based on the standards established by the Musculoskeletal Infection Society (MSIS) (Parvizi and Gehrke, 2014). The diagnosis of AL refers to the relevant criteria reported in previous literature (Huang et al., 2019), specifically including: (1) pain in the thigh or hip region, or knee pain; (2) radiographic evidence of prosthesis loosening, such as separation between the prosthesis components and bone tissue, displacement of the prosthesis components, or the presence of a radiolucent line; (3) negative periprosthetic culture; (4) exclusion of PJI.

**Table 1:** Musculoskeletal infection society criteria of the diagnosis of PJI.

Parameters	Number	MSIS Criteria
Major criteria Minor criteria	1 2 1 2 3 4 5	Two positive periprosthetic cultures with phenotypically identical organisms There is a sinus tract that communicates to the joint Elevated serum CRP (>10 mg/L) or ESR (>30 mm/h) Elevated synovial fluid white blood cell count (>3000 cells/ml) or changed positive leukocyte esterase strip test (++ or +++) Elevated synovial fluid percentage of granulocytes (>80%) A single positive culture Changed positive histologic analysis of the periprosthetic tissue (>5 neutrophils in each of the 5 high-power fields at 400 × magnification)

CRP, C-reactive protein; ESR, erythrocyte sedimentation rate. PJI is defined by the criteria of ≥f major or ≥r minor criteria.

### 2.3 Inclusion and exclusion criteria

Inclusion criteria: (1) Patients diagnosed with PJI or AL and undergoing corresponding treatment. (2) All study indicators for the patients are complete and available.

Exclusion criteria: (1) Patients with inflammatory diseases, such as rheumatoid arthritis, gout, systemic lupus erythematosus, etc. (2) Patients with cancer. (3) Patients with periprosthetic fractures. (4) Patients with prosthetic dislocation. (5) PJI occurring within 4 weeks after the initial total joint arthroplasty (acute PJI) (Zhang H and Wang K, 2021). (6) Abnormal liver function.

### 2.4 Statistical analysis

In this study, all statistical analyses were performed using IBM SPSS Statistics (version 21). For continuous variables, data were presented as mean ± standard deviation, while categorical variables were described using frequency (n) and percentage (%). In terms of statistical testing, the Mann-Whitney U test was used for continuous variables, whereas the Chi-square test or Fisher’s exact test was chosen for categorical variables based on the distribution of the data. A p-value of less than 0.05 was considered to indicate a statistically significant difference. Additionally, the diagnostic value of each biomarker was evaluated using receiver operating characteristic curves (ROC), area under the curve (AUC), and its 95% confidence interval (CI). The optimal cut-off value for each biomarker as a diagnostic tool for PJI was determined based on the Youden Index. Furthermore, the sensitivity, specificity, positive predictive value (PPV), and negative predictive value (NPV) of each biomarker were calculated to comprehensively assess their diagnostic performance. The DeLong’s test is used to compare the AUC values between biomarkers. Statistical significance: *P<0.05, **P<0.01, ***P<0.001.

## 3 Results

### 3.1 Demographic data

**Figure 1:**
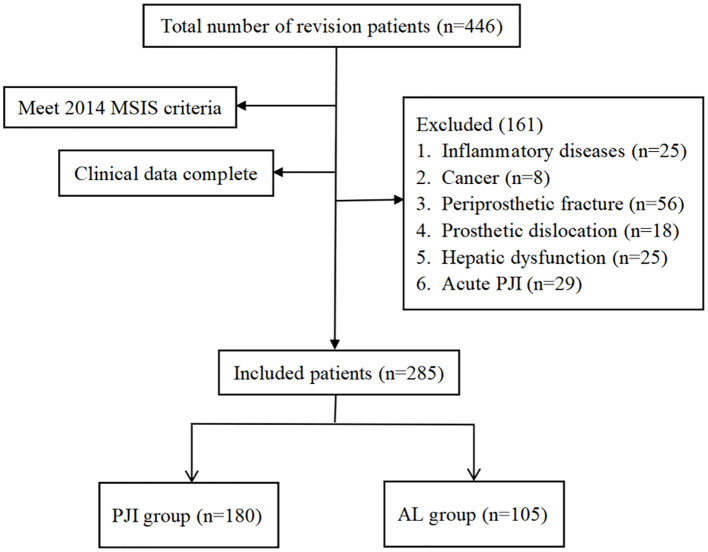
Flowchart of patient inclusion.

As shown in **Figure 1**, after screening, a total of 180 patients were included in the PJI group, while 105 patients were included in the AL group. As shown in **Table 2**, there were no significant differences between the two groups in terms of age, gender, and the prevalence of coronary heart disease (P>0.05). However, further analysis revealed that the prevalence of hypertension was significantly higher in the PJI group compared to the AL group (50.0%vs.37.1%, P=0.035). The prevalence of diabetes was also significantly higher in the PJI group than in the AL group (30.6%vs.17.1%, P=0.012). In addition, patients in the PJI group had significantly higher levels of CRP, ESR, and FIB compared to those in the AL group, while the PA level was significantly lower (P<0.001). Compared to patients with PJI, patients with AL were more likely to have abnormalities in the hip joint (P<0.001).

**Table 2:** Basic characteristics of the PJI group and the AL group.

Category	Entire Cohort (n=285)	PJI Group (n=180)	AL Group (n=105)	P value
Age (years)^†^ Gender^★^ Male Female Comorbidities^★^ Diabetes Hypertension CHD Joint^★^ Hip Knee	65.76 ± 11.17 114 (40.0) 171 (60.0) 73 (25.3) 129 (45.3) 50 (17.5) 154 (54.0) 131 (46.0)	66.20 ± 11.28 74 (41.1) 106 (58.9) 54 (30.0) 90 (50.0) 31 (17.2) 83 (46.1) 97 (53.9)	65.01 ± 10.98 40 (38.1) 65 (61.9) 18 (17.1) 39 (37.1) 19 (18.1) 71 (67.6) 34 (32.4)	0.386 0.616 0.016 0.035 0.852 <0.001
Markers^†^ CRP (mg/L) ESR (mm/h) FIB (g/L) PA (mg/L) CPR EPR FPR CEF	28.11 ± 45.68 48.09 ± 34.68 4.22 ± 1.98 194.62 ± 67.94 0.28 ± 0.70 0.49 ± 2.64 0.29 ± 0.30 0.80 ± 3.17	42.92 ± 51.94 64.49 ± 32.20 4.85 ± 1.65 167.26 ± 62.06 0.44 ± 0.84 0.72 ± 3.30 0.04 ± 0.03 1.20 ± 3.94	2.73 ± 4.93 19.96 ± 15.30 3.16 ± 2.06 241.52 ± 49.60 0.12 ± 0.25 0.09 ± 0.07 0.01 ± 0.01 0.11 ± 0.09	<0.001 <0.001 <0.001 <0.001 <0.001 <0.001 <0.001 <0.001

^†^The values are given as the mean and standard deviation; ^★^The values are given in terms of number of cases and percentages. P<0.05 was considered statistically significant. CHD, coronary heart disease; CRP, C-reactive protein; ESR, erythrocyte sedimentation rate; FIB, fibrinogen; PA, prealbumin; CPR, CRP/PA; EPR, ESR/PA; FPR, FIB/PA; CEF, CPR+EPR+FPR.

### 3.2 Levels of different markers in PJI group and AL group

**Figure 2:**
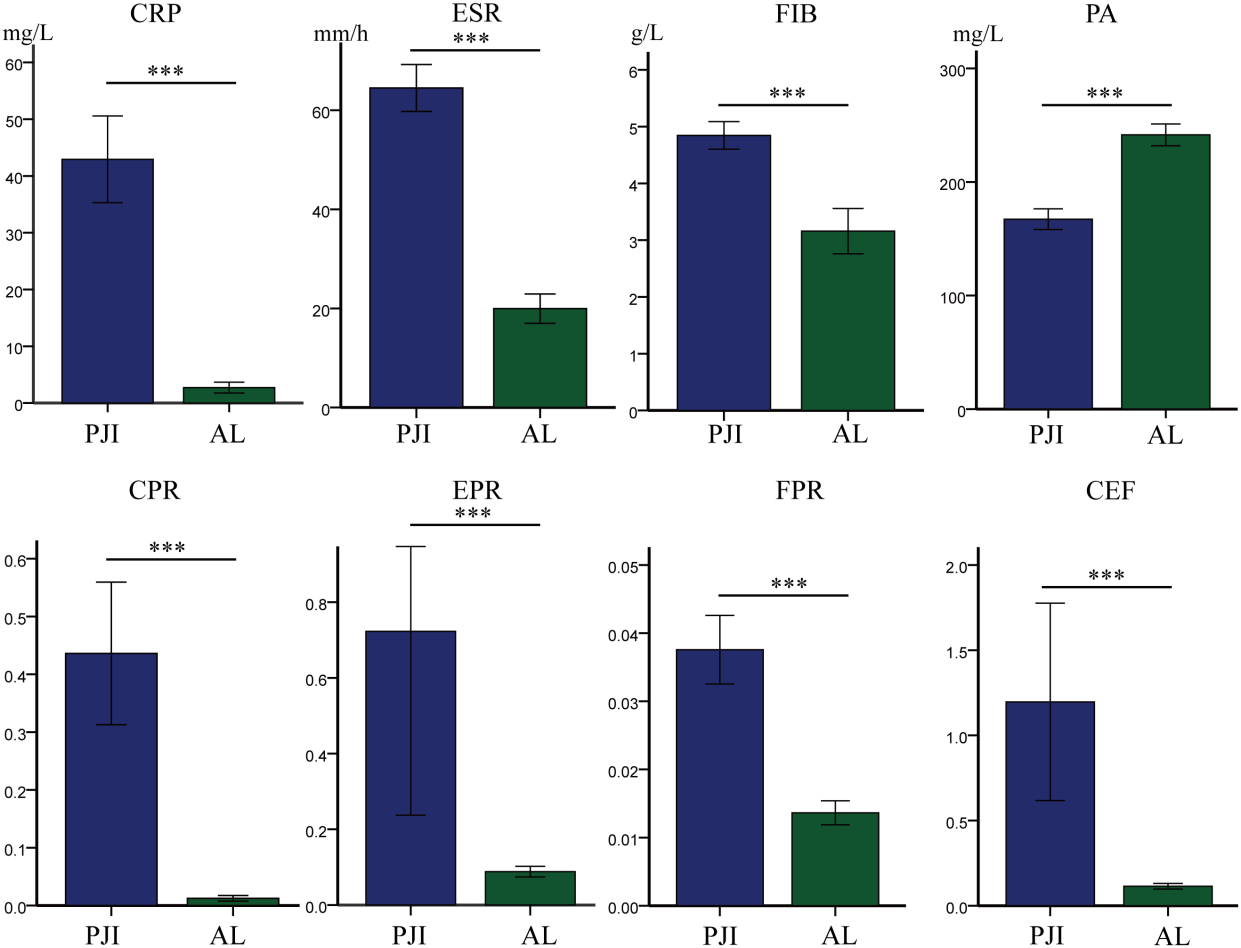
Comparison of levels of different markers between the PJI group and the AL group. Statistical significance: ***P<0.001. CRP, C-reactive protein; ESR, erythrocyte sedimentation rate; FIB, fibrinogen; PA, prealbumin; CPR, CRP/PA; EPR, ESR/PA; FPR, FIB/PA; CEF, CPR+EPR+FPR.

First, we compared the levels of traditional biomarkers between the PJI group and the AL group (**Table 2**, **Figure 2**). The results showed that the levels of CRP (42.92 ± 51.94 vs.2.73 ± 4.93, P<0.001), ESR (64.49 ± 32.20 vs.19.96 ± 15.30, P<0.001), and FIB (4.85 ± 1.65 vs.3.16 ± 2.06, P<0.001) were significantly higher in the PJI group than in the AL group. Subsequently, we compared the levels of PA, CPR, EPR, FPR, and CEF between the PJI group and the AL group (**Table 2**, **Figure 2**). The results indicated that the levels of CPR (0.44 ± 0.84 vs.0.12 ± 0.25, P<0.001), EPR (0.72 ± 3.30 vs.0.09 ± 0.07, P<0.001), FPR(0.04 ± 0.03 vs.0.01 ± 0.01, P<0.001), and CEF (1.20 ± 3.94 vs.0.11 ± 0.09, P<0.001) were significantly higher in the PJI group than in the AL group, while the level of PA (167.26 ± 62.06 vs.241.52 ± 49.60, P<0.001) was significantly lower in the PJI group than in the AL group.

### 3.3 The diagnostic value of different markers

**Figure 3:**
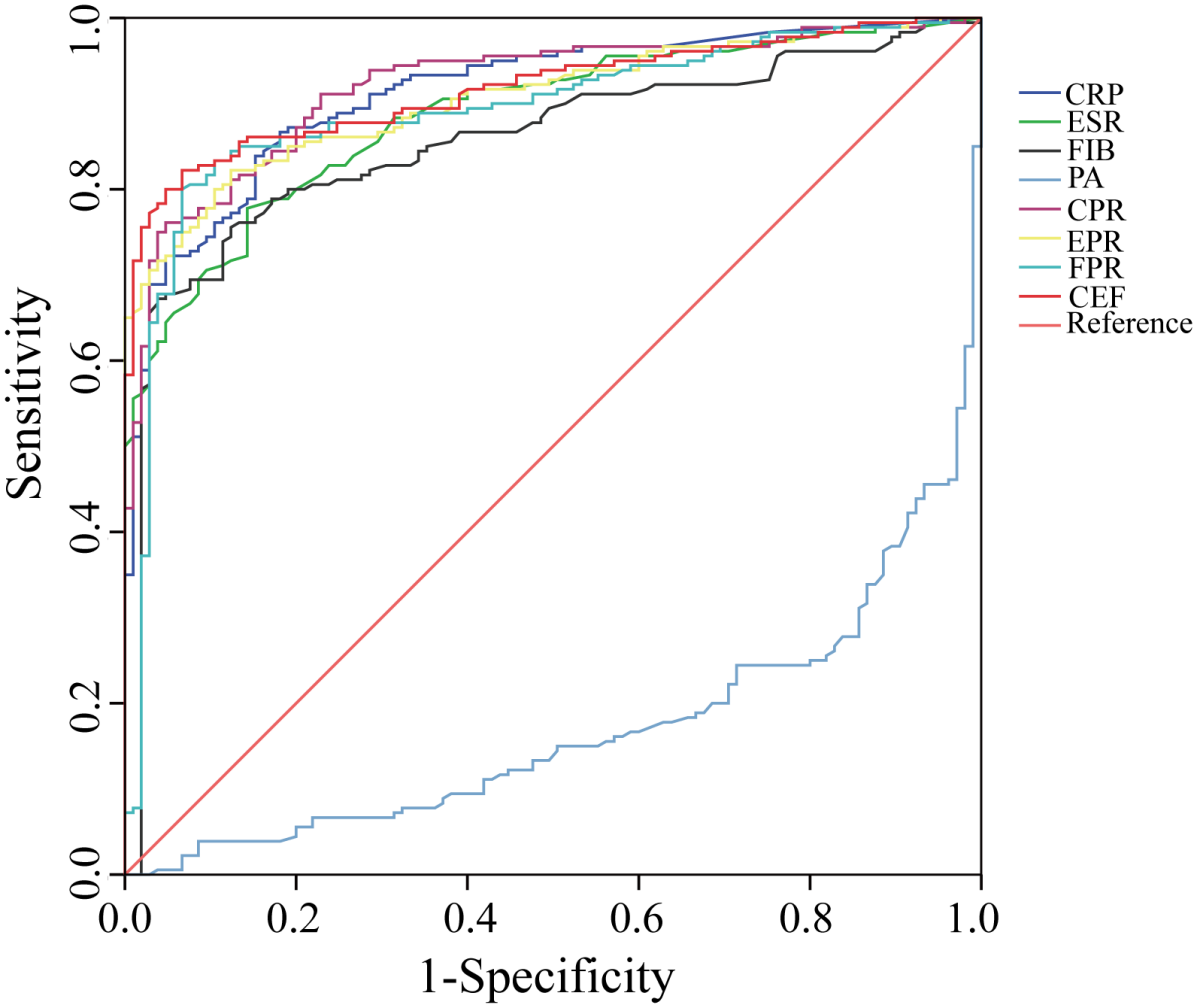
The ROC curves of CRP, ESR, FIB, PA, CPR, EPR, FPR, and CEF. CRP, C-reactive protein; ESR, erythrocyte sedimentation rate; FIB, fibrinogen; PA, prealbumin; CPR, CRP/PA; EPR, ESR/PA; FPR, FIB/PA; CEF, CPR+EPR+FPR.

We evaluated the ability of these biomarkers to distinguish PJI by calculating the AUC (**Table 3**, **Figure 3**). Among these biomarkers, CPR had the highest AUC of 0.921 (95%CI 0.890–0.952), and could distinguish PJI with a cutoff value of 0.0366, a sensitivity of 76.1%, and a specificity of 95.2%. To our delight, the ability of EPR and FPR to diagnose PJI was enhanced compared with that of ESR and FIB, respectively (AUC: 0.906 vs.0.887, 0.892 vs.0.855), and each biomarker had a strong ability to diagnose PJI (**Table 4**). Although PA had a low AUC of 0.169 (95%CI 0.123–0.216) and a poor ability to diagnose PJI, its specificity was as high as 96.2%. In addition, CEF showed a significant improvement in the diagnostic ability for PJI compared with EPR (**Table 4**).

**Table 3:** The diagnostic performance of different markers for PJI.

Markers	AUC	95%CI	Youden Index	Optimal Cutoff Value	Sen (%)	Spec (%)	PPV (%)	NPV (%)
CRP (mg/L)	0.913	(0.881-0.946)	0.687	9.543	83.9	84.8	91.46	75.21
ESR (mm/h)	0.887	(0.850-0.924)	0.635	36.500	77.8	85.7	89.92	58.97
FIB (g/L)	0.855	(0.809-0.901)	0.634	4.065	67.2	96.2	96.67	52.31
PA (mg/L) CPR EPR FPR CEF	0.169 0.921 0.906 0.892 0.918	(0.123-0.216) (0.890-0.952) (0.873-0.940) (0.852-0.932) (0.886-0.949)	-0.033 0.713 0.698 0.733 0.756	353.950 0.0366 0.1812 0.0181 0.2392	0.6 76.1 82.2 80.0 82.2	96.2 95.2 87.6 93.3 93.3	16.67 95.81 83.82 95.10 95.53	39.09 83.05 55.70 69.01 91.51

CI, confidence interval; CRP, C-reactive protein; ESR, erythrocyte sedimentation rate; FIB, fibrinogen; PA, prealbumin; CPR, CRP/PA; EPR, ESR/PA; FPR, FIB/PA; CEF, CPR+EPR+FPR; Sen, Sensitivity; Spec, Specificity; PPV, Positive Predictive Value; NPV, Negative Predictive Value.

**Table 4:** Pairwise comparison of ROC curves in PJI diagnosis.

Markers	Z value	P value
CRP-CPR ESR-EPR FIB-FPR CEF-CPR CEF-EPR CEF-FPR	-1.736 -2.875 -2.633 -0.279 2.260 1.838	0.083 0.004 0.008 0.780 0.024 0.066

CRP, C-reactive protein; ESR, erythrocyte sedimentation rate; FIB, fibrinogen; PA, prealbumin; CPR, CRP/PA; EPR, ESR/PA; FPR, FIB/PA; Statistical significance: P<0.05.

### 3.4 In the diabetes subgroups, the levels of different markers in the PJI group and the AL group

**Figure 4:**
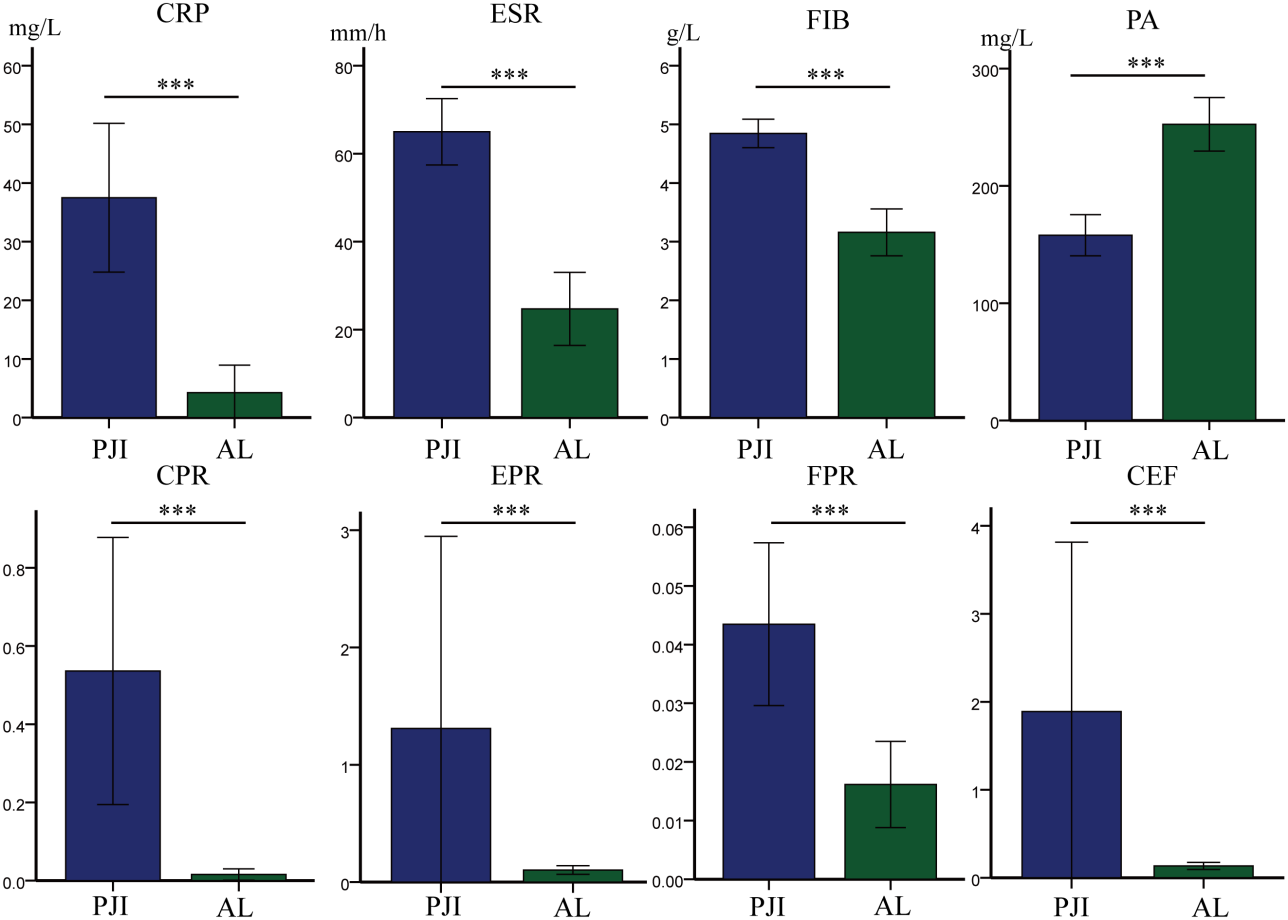
Among the diabetes subgroups, the levels of different markers in the PJI group and the AL group. CRP, C-reactive protein; ESR, erythrocyte sedimentation rate; FIB, fibrinogen; PA, prealbumin; CPR, CRP/PA; EPR, ESR/PA; FPR, FIB/PA; CEF, CPR+EPR+FPR. Statistical significance: ***P<0.001.

After further subgroup analysis of all patients with PJI and AL, we found that among patients with diabetes, the PJI group had significantly higher levels of CRP (37.49 ± 46.47 vs.4.25 ± 9.42, p<0.001), ESR (64.98 ± 27.64 vs.24.72 ± 16.70, p<0.001), FIB (4.66 ± 1.55 vs.3.92 ± 3.44, p<0.001), CPR (0.54 ± 1.25 vs.0.02 ± 0.03, p<0.001), EPR (1.31 ± 6.00 vs.0.10 ± 0.74, p<0.001), FPR (0.04 ± 0.05 vs.0.02 ± 0.01, p<0.001), and CEF(1.89 ± 706 vs.0.13 ± 0.80, p<0.001) compared to the AL group (**Table 5**, **Figure 4**). Conversely, the PJI group had significantly lower levels of PA (157.93 ± 64.31 vs.252.47 ± 45.93, p<0.001) compared to the AL group among patients with diabetes.

**Table 5:** Among the diabetes subgroups, the levels of different markers in the PJI group and the AL group.

Markers	PJI Group(n=54)	AL Group (n=18)	P value
CRP(mg/L) ESR(mm/h) FIB(g/L) PA(mg/L) CPR EPR FPR CEF	37.49 ± 46.47 64.98 ± 27.64 4.66 ± 1.55 157.93 ± 64.31 0.54 ± 1.25 1.31 ± 6.00 0.04 ± 0.05 1.89 ± 0.71	4.25 ± 9.42 24.72 ± 16.70 3.92 ± 3.44 252.47 ± 45.93 0.02 ± 0.03 0.10 ± 0.74 0.02 ± 0.01 0.13 ± 0.80	<0.001 <0.001 <0.001 <0.001 <0.001 <0.001 <0.001 <0.001

CRP, C-reactive protein; ESR, erythrocyte sedimentation rate; FIB, fibrinogen; PA, prealbumin; CPR, CRP/PA; EPR, ESR/PA; FPR, FIB/PA; CEF, CPR+EPR+FPR.

### 3.5 In the diabetes subgroups, the diagnostic value of different markers

**Figure 5:**
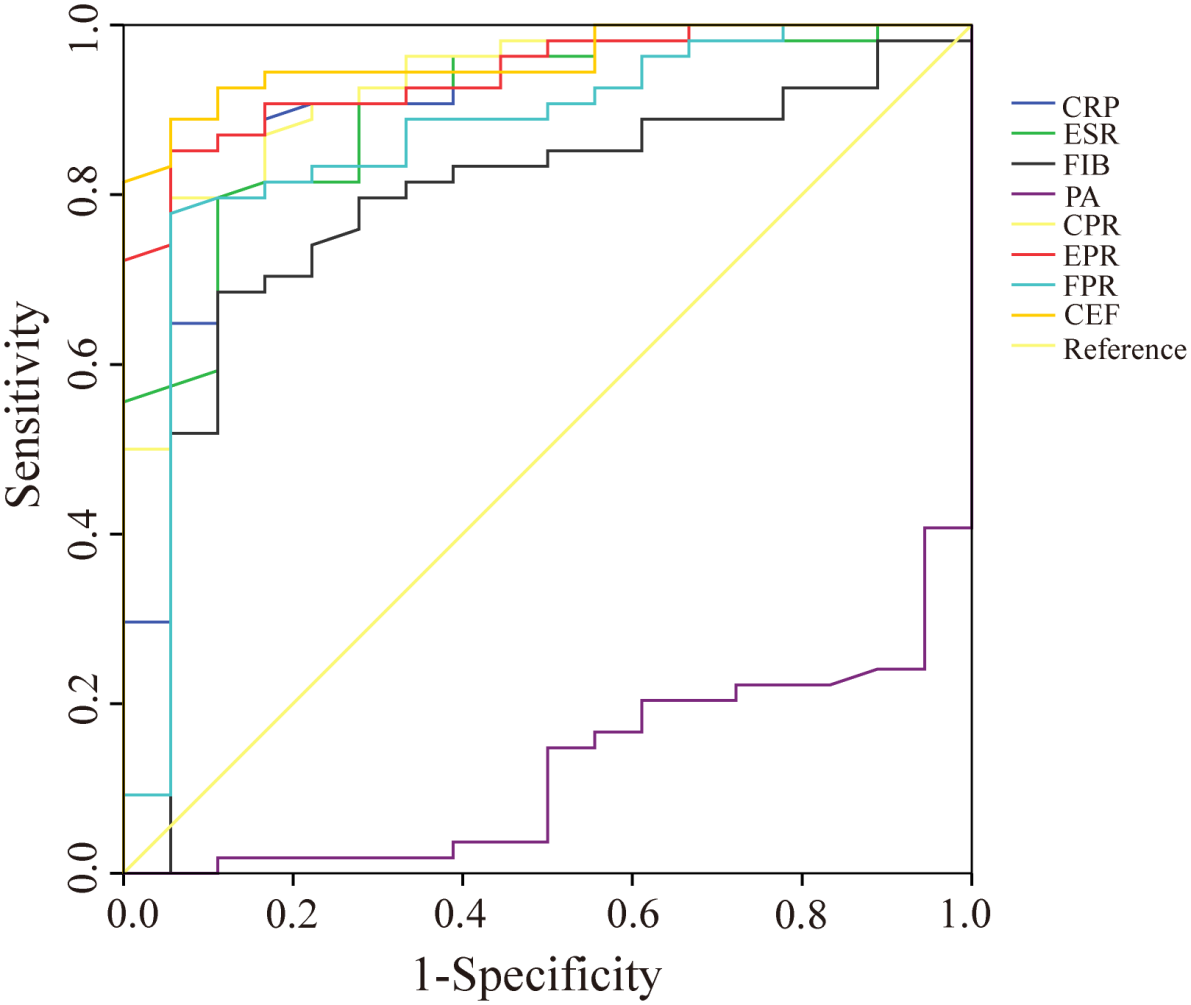
The ROC curves of CRP, ESR, FIB, PA, CPR, EPR, FPR, and CEF in the diabetes subgroups. CRP, C-reactive protein; ESR, erythrocyte sedimentation rate; FIB, fibrinogen; PA, prealbumin; CPR, CRP/PA; EPR, ESR/PA; FPR, FIB/PA; CEF, CPR+EPR+FPR.

In the diabetic subgroup, the ROC curve analysis showed that the highest AUC value for CEF was 0.958 (95%CI 0.917–1.000), and it could identify PJI with a cutoff value of 0.833, a sensitivity of 88.9%, and a specificity of 94.4% (**Table 6**, **Figure 5**). Compared with traditional markers (CRP, ESR), the markers combined with PA (CPR, EPR) showed no significant improvement in the diagnostic ability for PJI in diabetic patients (AUC: 0.904 vs. 0.927, 0.900 vs. 0.941) (**Table 7**). In contrast to our research on all PJI patients, in the subgroup analysis, we found that CEF had no significant difference in diagnostic ability for PJI in diabetic patients compared with CPR, EPR, and FPR (**Table 7**).

**Table 6:** Diagnostic value of different markers in the diabetes subgroups.

Markers	AUC	95% CI	Youden Index	Optimal Cutoff Value	Sen(%)	Spec(%)	PPV(%)	NPV(%)
CRP(mg/L)	0.904	(0.817-0.991)	0.722	8.885	88.9	83.3	94.55	88.24
ESR(mm/h)	0.900	(0.823-0.976)	0.685	43.000	79.6	88.9	95.56	59.26
FIB(g/L)	0.784	(0.660-0.909)	0.574	3.920	68.5	88.9	94.29	43.24
PA(mg/L) CPR EPR FPR CEF	0.123 0.927 0.941 0.866 0.958	(0.044-0.202) (0.861-0.994) (0.890-0.992) (0.759-0.972) (0.917-1.000)	-0.092 0.741 0.796 0.722 0.833	298.500 0.375 0.203 0.018 0.231	1.9 79.6 85.2 77.8 88.9	88.9 94.4 94.4 94.4 94.4	66.67 1.00 97.96 97.37 97.73	24.24 30.00 73.91 50.00 60.71

CI, confidence interval; CRP, C-reactive protein; ESR, erythrocyte sedimentation rate; FIB, fibrinogen; PA, prealbumin; CPR, CRP/PA; EPR, ESR/PA; FPR, FIB/PA; CEF, CPR+EPR+FPR; Sen, Sensitivity; Spec, Specificity; PPV, Positive Predictive Value; NPV, Negative Predictive Value.

**Table 7:** Pairwise comparison of ROC curves in the diabetic PJI subgroup.

Markers	Z value	P value
CRP-CPR ESR-EPR FIB-FPR CEF-CPR CEF-EPR CEF-FPR	-1.598 -1.948 -2.493 0.982 1.150 1.813	0.110 0.051 0.013 0.326 0.250 0.070

CRP, C-reactive protein; ESR, erythrocyte sedimentation rate; FIB, fibrinogen; PA, prealbumin; CPR, CRP/PA; EPR, ESR/PA; FPR, FIB/PA; Statistical significance: P<0.05.

## 4 Discussion

Despite the availability of multiple biomarkers for predicting PJI, its timely and accurate diagnosis still faces many challenges. This study is the first to explore the value of PA and its related ratios (CPR, EPR, and FPR) in the diagnosis of PJI. The results show that compared with traditional markers (CRP, ESR, and FIB), these three new ratio-based markers (CPR, EPR, and FPR) not only demonstrate superior diagnostic value but also have higher sensitivity and specificity. In addition, this study found that the prevalence of diabetes in the PJI group was significantly higher than that in the AL group. Therefore, we further analyzed the diagnostic efficacy of PA, CPR, EPR, and FPR in PJI patients with diabetes. Excitingly, the combined index CEF achieved an AUC value as high as 0.951 in the diagnosis of PJI. Our study results indicate that CPR, EPR, and FPR can serve as powerful auxiliary tools for the diagnosis of PJI, while CEF can be used to assist in the diagnosis of PJI in patients with diabetes. It is worth noting that although the PA levels in the PJI group were significantly lower than those in the AL group, PA itself does not have the ability to independently diagnose PJI. Since the markers used in this study are all from the routine serum tests of inpatients, they have low cost and high accessibility.

### 4.1 The diagnostic value of CPR for PJI

CRP is an acute-phase protein synthesized by the liver (Marnell et al., 2005). Its levels significantly increase in the presence of inflammation, infection, or other types of tissue damage, making it an important biomarker for the body’s inflammatory response (Pieri et al., 2014). Multiple studies have reported that the ratios of CRP to other biomarkers show better diagnostic capabilities for PJI (Shi et al., 2021; Luger et al., 2024). However, these CRP-based ratio indicators do not significantly outperform CRP itself in terms of sensitivity and specificity for diagnosing PJI (Christopher et al., 2021; Jiao et al., 2022). Previous studies have demonstrated that CPR levels are positively correlated with the activity of rheumatoid arthritis, suggesting its potential as a novel inflammatory biomarker (Wang et al., 2020). However, to our knowledge, no studies have yet explored the diagnostic efficacy of CPR in PJI. In our study, we found that the specificity of CPR for diagnosing PJI was as high as 95.2%. Additionally, in the diabetic subgroup, the specificity of CPR reached as high as 94.4%, showing a significant advantage. The AUC value of CPR was also as high as 0.927, which further highlights its diagnostic efficacy in this subgroup. These results indicate that CPR is a promising biomarker for diagnosing PJI and has the potential to greatly reduce the misdiagnosis rate of PJI.

### 4.2 The diagnostic value of PA and EPR for PJI

PA is synthesized in the liver and is not only an indicator for assessing patients’ nutritional status but also closely related to inflammation, infection, and tumor diseases (Ang et al., 1991; Shenkin, 2006; Hrnciarikova et al., 2007). Our study results showed that the PA level in the PJI group was significantly lower than that in the AL group, which is consistent with the results of other inflammatory-related diseases (Song et al., 2024; Jin et al., 2025). We speculate that this result may be related to the poor nutritional status and inflammatory state of PJI patients. At the same time, our study revealed that PA does not have the ability to diagnose PJI, with an AUC value of only 0.169. Currently, ESR has been used as a first-line screening marker for PJI diagnosis. However, the limitation of ESR is that its level is affected by systemic inflammation and infectious diseases (Shahi et al., 2015). To our knowledge, no studies have explored the value of EPR in diseases so far. Yet, our research found that EPR has a good ability to diagnose PJI, with specificity and sensitivity of 87.6% and 82.2%, respectively. In the diabetes subgroup, we were pleasantly surprised to find that EPR performed well in diagnosing PJI in diabetic patients. Its AUC value was as high as 0.941, with sensitivity reaching 85.2% and specificity as high as 94.4%. These results indicate that EPR has a significant advantage in the accuracy of diagnosing PJI in diabetic patients, providing strong support for clinical diagnosis. The above research results suggest that EPR may be a highly potential inflammatory marker, which is worth further exploration.

### 4.3 The diagnostic value of FIB and FPR for PJI

FIB is a plasma glycoprotein synthesized by the liver and serves as coagulation factor I, playing a key role in the coagulation process (Singh et al., 2025). Numerous studies have reported that FIB is a promising biomarker for the diagnosis of PJI before revision arthroplasty (Li et al., 2019; Xu et al., 2020; Xu et al., 2022). Our research results show that, with an optimal cutoff value of 4.065 g/L, FIB can serve as a sensitive biomarker for diagnosing PJI in patients undergoing revision arthroplasty (with a specificity of 96.2%). This optimal cutoff value is similar to that reported in a previous study for diagnosing PJI after primary joint arthroplasty (4.01 g/L) (Li et al., 2019). However, in the analysis of the diabetes subgroup, the diagnostic capability of FIB was relatively poor (AUC:0.784, sensitivity:68.5%, specificity:88.9%).

Previous studies have found that preoperative FPR is a promising marker for predicting the clinical prognosis of patients with stage II-III colorectal and gastric cancer (Zhang et al., 2017; Sun et al., 2018). Additionally, it has been reported that patients with stroke have higher FPR levels, and FPR is closely associated with stroke-related pneumonia (Qiu et al., 2024). Therefore, FPR is expected to become a new inflammatory marker. To date, no studies have explored the efficacy of FPR in diagnosing PJI. Our study found that FPR has a high value in diagnosing PJI (AUC:0.892), with a specificity of 93.3% for PJI diagnosis, which is higher than that of CRP (84.8%). In the diabetes subgroup, the efficacy of FPR in diagnosing PJI slightly decreased, but its specificity for PJI diagnosis slightly increased. These results indicate that FPR is a promising marker for diagnosing PJI and can significantly reduce the misdiagnosis rate of PJI.

### 4.4 The diagnostic value of CEF for PJI

Previous studies have reported that combining multiple biomarkers can enhance the diagnostic efficacy for PJI (Qin et al., 2020; Huang et al., 2021; Maimaiti et al., 2022). Therefore, we combined three biomarkers with excellent diagnostic performance (CPR, EPR, and FPR) into a composite biomarker called CEF to investigate its diagnostic capability for PJI. However, the results showed that the diagnostic ability of CEF did not significantly improve. A large number of studies have reported that diabetes can increase the incidence of PJI by delaying wound healing and impairing the immune system (Blanco et al., 2020; Iannotti et al., 2020; Rodriguez-Merchan and Delgado-Martinez, 2022). Our study results indicated that, compared with the AL group, the prevalence of diabetes in the PJI group significantly increased, reaching 30.0%. In the diabetes subgroup, CEF demonstrated strong diagnostic ability among various biomarkers, with an AUC of 0.958. Moreover, the sensitivity and specificity of CEF for diagnosing PJI in patients with diabetes were 88.9% and 94.4%, respectively. These findings demonstrate the diagnostic value of CEF in this specific subgroup and suggest that it has the potential to become an important tool for the precise diagnosis of diabetes-related PJI. However, the limited sample size in the analysis of the diabetes subgroup may have somewhat affected the accuracy of the results.

### 4.5 Limitations

The present study has the following limitations: (1) This study is a retrospective study, which may have inherent biases; (2) This study is a single-center study with a relatively small number of patients included in the subgroup analysis, which may lead to underpowered analyses; (3) The patients included in this study are of a single race, and caution is needed when extending the findings to other races; (4) This study did not adjust for the influence of other confounding factors, which may affect the accuracy of the results. Therefore, in the future, we will adopt a prospective, multi-center, multi-ethnic, large-sample study to further validate the results of this study. Meanwhile, in future studies, we will use logistic regression to adjust for confounding factors such as age, diabetes, and hypertension, and explore whether these confounding factors affect the diagnostic accuracy of biomarkers for PJI. In the future, we will also conduct a larger sample size analysis of the diabetes subgroup to address the issue of underpowered analyses. Finally, in the future, we will also conduct cost-effectiveness analyses and comparative studies of the effectiveness of emerging diagnostic methods.

## 5 Conclusion

In summary, CPR, EPR, and FPR, as promising markers, can be used for the diagnosis of PJI. Meanwhile, CEF is a potential marker for the diagnosis of PJI with diabetes. However, due to their relatively low sensitivity, it is recommended to use them in combination with other markers. Nevertheless, further comprehensive studies are still needed to verify and refine this combined diagnostic approach.

## Data Availability

The original contributions presented in the study are included in the article/supplementary material. Further inquiries can be directed to the corresponding authors.
